# Hepatitis B Seroprevalence and Associated Factors Among Inpatients from 2014 to 2023: A Single-Centre Retrospective Study in a Malaysian Tertiary Hospital

**DOI:** 10.21315/mjms-04-2025-240

**Published:** 2026-02-28

**Authors:** Nurul Asyikin Kamaluddin, Nurzulaikha Abdullah, Yeong Yeh Lee, Mung Seong Wong, Nani Draman, Nazri Mustaffa

**Affiliations:** 1 Department of Internal Medicine, School of Medical Sciences, Universiti Sains Malaysia, Health Campus, Kelantan, Malaysia; 2 Hospital Pakar Universiti Sains Malaysia, Universiti Sains Malaysia, Health Campus, Kelantan, Malaysia; 3 Faculty of Data Science and Computing, Universiti Malaysia Kelantan, Pengkalan Chepa, Kelantan, Malaysia; 4 Department of Medicine, Faculty of Medicine and Health Sciences, Universiti Putra Malaysia, Serdang, Selangor, Malaysia; 5 Department of Medicine, Hospital Sultan Abdul Aziz Shah, Universiti Putra Malaysia, Serdang, Selangor, Malaysia; 6 Department of Family Medicine, School of Medical Sciences, Universiti Sains Malaysia, Health Campus, Kelantan, Malaysia

**Keywords:** Hepatitis B virus, HBsAg, seroprevalence, Malaysia

## Abstract

**Background:**

Hepatitis B virus (HBV) remains a global health burden, causing significant health problems, including morbidity and mortality. The World Health Organization (WHO) aims to reduce new infections by 90% and deaths by 65% by 2030, with a focus on the Western Pacific and Southeast Asia. In Malaysia, HBV incidence rose from 2.26 per 100,000 in 2010 to 12.65 per 100,000 in 2015, classifying the country as having an intermediate burden with a seroprevalence of 1.5% to 9.8%. The major transmission routes include perinatal transmission, blood exposure, sexual contact, and needle sharing. This study aimed to provide insight into the epidemiology and disease burden of HBV in a large tertiary hospital in Malaysia. Insights from this study will assist in future directions regarding public health strategies to reduce the burden of hepatitis B by 2030.

**Methods:**

This study investigated the seroprevalence of hepatitis B infection (HBsAg) among inpatients at Hospital Universiti Sains Malaysia over 10 years from 1 January 2014 to 31 December 2023. This retrospective study included patients who underwent HBsAg screening during ward admission. Subjects were selected based on probability sampling after reviewing the inclusion and exclusion criteria. Descriptive and inferential analyses were performed following data collection. Statistical significance was determined by *P*-values using IBM SPSS version 29.

**Results:**

A total of 308 patients were identified. The overall seroprevalence was 9.1% (95% CI: 0.06, 0.12), with annual rates fluctuating between 6.3% and 10.7%. The highest positivity rate was observed in the 45 to 54 age group (14.3%), whereas the lowest positivity rate was observed in those aged > 64 years (5.0%). Males exhibited a higher positivity rate (11.7%) than females (6.5%). Private employees had the highest positivity rate (28.6%) among the occupational groups. Individuals coinfected with HIV had a strikingly high positivity rate of 62.5%. Our analysis identified needle sharing as a significant risk factor, with an adjusted odds ratio of (OR = 8.87; 95% CI: 1.64, 47.91; *P* = 0.011). Other risk factors, such as family history of chronic hepatitis, multiple sexual partners, history of blood transfusions, and body tattooing, showed increased odds but were not statistically significant.

**Conclusion:**

Our study provides insights into the local epidemiology, the burden of hepatitis B infection, and the factors that contribute to disease transmission. This study highlights the need for targeted public health interventions to reduce risk behaviours, particularly needle sharing, to control the spread of hepatitis B in the region.

## Introduction

Hepatitis B virus (HBV) infection is a worldwide burden to the healthcare system, causing significant morbidity and mortality. Hepatitis B contributes to an estimated 820,000 deaths per year globally (1). The epidemiology of hepatitis B infection is changing mainly due to migration and vaccination factors. The World Health Organization (WHO) reported that the highest hepatitis B infection rates are in Western Pacific countries, with an estimated 116 million chronically infected people. An estimated 18 million people are infected with HBV in the Southeast Asian region (2). In 2016, the WHO launched its global strategy to combat hepatitis B and C, aiming for elimination by 2030. The strategy’s goals include reducing new hepatitis infections by 90% and hepatitis-related deaths by 65% between 2016 and 2030 (3).

In Malaysia, HBV infection has raised public health concerns with an upward trend from 2.26 per 100,000 in 2010 to 12.65 per 100,000 in 2015 (4). Malaysia has an intermediate burden of HBV infection, with a high prevalence rate, with seroprevalence ranging from 1.5% to 9.8% (5). End sequelae of viral hepatitis, including cirrhosis and liver cancer, were the sixth major cause of death in Malaysia between 2013 and 2015 (4). In Malaysia, mandatory notification for HBV diagnosis was introduced in 2010 under the First Schedule of the Control and Prevention of Communicable Diseases Act 1988 (6). The Ministry of Health of Malaysia also developed the National Strategic Plan for Hepatitis B and C task force in 2019 to combat viral hepatitis by 2030.

Approximately one million Malaysians are chronically infected with HBV (4). The significant route of HBV transmission is perinatal transmission from the fetus through infected mother-to-child transmission at birth and horizontal transmission from exposure to infected blood (1). Other transmission routes include sexual transmission, needle sharing or needlestick injury, tattooing, piercing, or exposure to infected fluids such as saliva, vaginal, or seminal fluids. Hepatitis B infection during infancy and early childhood before the age of five years predisposes individuals to a higher risk of developing chronic hepatitis in approximately 95% of cases. In contrast, most cases of HBV infection acquired in adulthood resolve on their own, with fewer than 5% progressing to chronic hepatitis (3).

The disease severity varies, ranging from asymptomatic or subclinical infection to symptomatic hepatitis and, in some cases, severe fulminant hepatitis leading to liver failure (7). Patients may have typical symptoms in symptomatic cases, including anorexia, malaise, and jaundice. Some patients may develop severe acute hepatitis B infection characterised by coagulopathy with an INR of > 1.5, or a protracted course (i.e., persistent symptoms or marked jaundice for > 4 weeks) or signs of acute liver failure (8). Chronic hepatitis B is defined as the presence of detectable hepatitis B surface antigen (HBsAg) in the blood or serum for longer than six months (9). Chronic hepatitis B can be classified into five phases: HBeAg-positive chronic infection, HBeAg-positive chronic hepatitis, HBeAg-negative chronic infection, HBeAg-negative chronic hepatitis, and HBsAg-negative phase (8). The presence of e antigen (HBeAg) is associated with a higher rate of viral replication and infectivity (10).

Individuals with chronic hepatitis B infection may remain asymptomatic while the infection remains inactive, without significant health problems. However, others may progress to chronic liver disease complications such as liver fibrosis, cirrhosis, or hepatocellular carcinoma (11). Without antiviral treatment, patients with cirrhosis face a significant risk of developing decompensated cirrhosis. If left untreated, the five-year survival rate of patients with decompensated cirrhosis can be as low as 15% (12). The primary goal of antiviral therapy is to enhance survival and quality of life by halting disease progression and preventing the development of hepatocellular carcinoma. Current evidence-based guidelines recommend initiating antiviral treatment when the HBV DNA level exceeds 2,000 IU/mL, the ALT level is elevated, or moderate or more significant histological lesions are present. In addition, all patients with cirrhosis with detectable HBV DNA should receive treatment (8).

Currently, there are two main treatment options for chronic hepatitis B: nucleoside or nucleotide analogues (NAs) or pegylated interferon α: (PEG-IFN-α). Examples of NAs that have been approved for use in hepatitis B include lamivudine, adefovir dipivoxil, entecavir, telbivudine, tenofovir disoproxil fumarate, and tenofovir alafenamide. However, the current preferred treatment of choice for long-term administration is potent NAs with a high barrier to resistance: entecavir, tenofovir disoproxil fumarate, or alafenamide. After starting with antiviral treatment, the primary endpoint of therapy is the induction of long-term HBV DNA suppression. Other therapy endpoints include HBeAg loss induction, ALT normalisation, and HBsAg loss with or without anti-HBs seroconversion (8).

Therefore, the current study provides insights into the epidemiology, characteristics, and disease burden of HBV at Hospital Pakar Universiti Sains Malaysia, a large public university hospital located on the east coast of Peninsular Malaysia. This study will contribute to existing knowledge on the nationwide disease burden that can be used to design targeted public health interventions.

## Methods

### Study Population and Design

This retrospective cross-sectional study was conducted at Hospital Pakar Universiti Sains Malaysia (HPUSM), a tertiary referral hospital in Kelantan, Malaysia. The study analysed hospital records over 10 years, from 1 January 2014 to 31 December 2023, involving patients who underwent screening tests for HBsAg at HPUSM. The study population comprised adult inpatients (aged ≥ 18 years) who underwent screening for hepatitis B surface antigen (HBsAg) during hospital admission.

All inpatients who underwent an HBsAg test upon admission were identified using the online hospital database. The inclusion criteria were adult inpatients aged 18 years or older who underwent HBsAg testing upon hospital admission and had complete demographic, clinical, and laboratory data. The exclusion criteria were as follows: incomplete or missing HBsAg results, incomplete demographic, clinical, and laboratory data; or repeat admissions during the study period. Using probability sampling, 308 subjects were finally selected after reviewing the inclusion and exclusion criteria.

### Statistical Analysis

For the descriptive analysis, the categorical variables were summarised using frequency and percentage, while the mean and standard deviation of the numerical variables were described if they followed a normal distribution. For the inferential analysis, both simple and multiple logistic regression models were used to determine the association between independent variables, with hepatitis B as the dependent variable. A simple logistic regression was initially conducted for each predictor variable to identify potential associations. Variables with a *P*-value of 0.25 in the univariate analysis were subsequently included in the multiple logistic regression model to adjust for potential confounding factors and determine the independent effect of each predictor. Statistical significance for each factor was evaluated using *P*-values, with a significance threshold set at *P* < 0.05. The strength of associations was expressed as odds ratios with 95% confidence intervals. All statistical analyses were performed using the Statistical Package for the Social Sciences software (version 29).

### Ethical

This study complied with the Declaration of Helsinki’s ethical principles and the Malaysian Good Clinical Practice Guidelines. The study was approved by the Human Ethics Research Committee of Universiti Sains Malaysia (study protocol code USM/JEPeM/KK/24040298). Data confidentiality was maintained at the highest level possible; only researchers had access to the data. Information was recorded under an anonymised subject number and other subject identification codes.

## Results

Using HPUSM ward admission data from 1 January 2014 to 31 December 2023, 54,445 HBsAg tests were requested. This was reduced to 22,773 tests after duplicate data removal. Following simple random sampling, 308 patients were identified and selected for further data retrieval, with each year contributing between 9.1% and 10.7% of the total sample.

**Figure 1:**
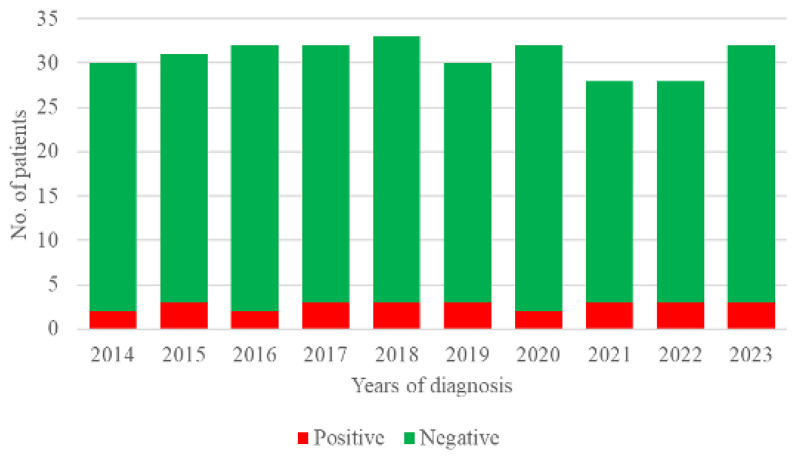
Yearly distribution of HBsAg status from 2014 to 2023

The details of the distribution of patients categorised according to their HBsAg status are outlined in Table 1. The overall seroprevalence of HBsAg across these years was 9.1% (95% CI: 0.06, 0.12), with 28 of 308 patients testing positive. The data from this study suggest a 95% confidence that the true seroprevalence of HBsAg in the broader population could range from 6% to 12%. The annual seroprevalence fluctuated slightly, ranging from 6.3% to 10.7%, with no significant upward or downward trend observed over the period (Figure 1). Most patients, accounting for 90.9%, were negative for HBsAg, indicating a relatively low seroprevalence across 10 years within the studied population. These findings highlight the stability of HBsAg seroprevalence in the population over time, with consistent negative rates exceeding 89.0% each year.

### Sociodemographic Analysis of Patients Who Underwent HBsAg Screening at HPUSM

This study found that patients aged 45 years constituted the largest age group (40.6% of the total population). However, the highest HBsAg positivity rate was observed in the 45 to 54 age group, with 14.3% testing positive. In contrast, the lowest HBsAg positivity rate was observed among patients aged > 64 years, with only 5.0% testing positive. The distribution of males and females was equal, with each group comprising 50.0% of the population. Males had a higher HBsAg positivity rate of 11.7% than females (6.5%). Most patients were Malay (92.2%); within this group, 9.2% tested positive for HBsAg. The analysis revealed that private employees comprised only 6.8% of the total population and had the highest HBsAg positivity rate at 28.6%, followed by self-employed individuals at 11.5%. Among marital status categories, divorcees represented only 3.5% of the population but had the highest HBsAg positivity rate at 27.3%.

The analysis of coinfection status revealed that participants who were coinfected with hepatitis C, who made up 4.5% of the total population, had an HBsAg positivity rate of 21.4%. In comparison, those coinfected with HIV, comprising just 2.6% of the population, exhibited a higher positivity rate of 62.5%. Participants with Type 2 diabetes mellitus (T2DM), representing 32.5% of the total population, had an HBsAg positivity rate of 8.0%. Patients with hypertension, who made up 42.2% of the population, had a positivity rate of 7.7%. Participants with hyperlipidemia, which accounted for 26.0% of the population, had a positivity rate of 8.8%.

In contrast, those with obesity, comprising 4.9% of the total population, had the lowest positivity rate among the comorbidities examined (6.7%). These findings highlight the varying degrees of HBsAg positivity across different coinfection and comorbidity groups within the study population. Table 2 shows the distribution of HBsAg status across various demographic and medical history factors.

The analysis of family and behavioural risk factors further highlighted that although these specific risk factors represent only a tiny portion of the overall study population, they exhibit higher HBsAg positivity rates. Among participants with a family history of chronic hepatitis and those with a history of body tattoos, who constituted 2.6% and 0.3% of the total study population, respectively, the HBsAg positivity rate was strikingly high at 100.0% in both groups. Participants with a history of receiving blood transfusions, who made up 8.8% of the population, had an HBsAg positivity rate of 14.8%. Similarly, those with a history of sharing needles (representing only 1.9% of the study population) had a significantly high positivity rate of 50.0%. Regarding sexual behaviour, participants with multiple sexual partners, comprising 8.8% of the study population, had a positivity rate of 14.8%.

### Factors Associated with Acquired Hepatitis B Infection

Simple logistic regression analysis was used to examine the association between various factors and the likelihood of hepatitis B infection before proceeding to multiple logistic regression (Table 3). Similar to the sociodemographic factor analysis, this study shows that female patients had 0.53 times the odds of being seropositive for hepatitis B as compared to males (OR = 0.53; 95% CI: 0.23, 1.18; *P* = 0.118), suggesting a 47% lower odds of infection, whereas non-Malay patients had 11% higher odds of infection (OR = 1.11; 95% CI: 0.25, 4.98; *P* = 0.893) compared to Malays. However, neither of these associations was significant.

This study also examined the association between medical history and risk behaviour factors with hepatitis B infection. Patients with a history of receiving blood transfusion had 86% higher odds of hepatitis B infection (OR = 1.86; 95% CI: 0.60, 5.83; *P* = 0.286) than those without such a history. Patients with multiple sexual partners also had 86% higher odds of being seropositive for hepatitis B (OR = 1.86; 95% CI: 0.59, 5.81; *P* = 0.289) than those without multiple partners. However, these two associations were also not statistically significant. Finally, patients who had practiced needle sharing were associated with statistically significantly higher odds of infection (OR = 11.08; 95% CI: 2.12, 57.80; *P* = 0.004), with more than 11 times the odds of hepatitis B infection compared to those who did not share needles.

Table 3 summarises the conducted logistic regression analyses. Two variables, gender and needle sharing, with *P* <0.25, were selected for inclusion in the multiple logistic regression analysis.

Multiple logistic regression was performed using the standard (forced entry) method to identify factors associated with HBV. All selected independent variables were simultaneously entered into the model using this method. This approach allows the independent effect of each factor to be assessed while controlling for the other variables in the model. The initial analysis revealed that needle sharing was associated with more than 11 times the odds of hepatitis B infection compared with those who did not share needles. Even after adjusting for the other variables, needle sharing remained a significant predictor of hepatitis B seroprevalence, although the odds decreased (Adj. OR = 8.87; 95% CI: 1.64, 47.91; *P* = 0.011) (Table 4). This adjusted odds ratio corresponds to an approximately 787% increased likelihood of HBV infection among patients who practiced needle sharing compared with those who did not, even when controlling for sex.

However, the initial analysis found that female patients had 47% lower odds of being seropositive for hepatitis B than male patients, although this difference was not statistically significant. In the final model of the multiple logistic regression analysis, the odds ratio changed only slightly after adjusting for needle sharing. However, gender differences in hepatitis B seroprevalence remained statistically nonsignificant (Adj. OR = 0.62; 95% CI: 0.27, 1.42; *P* = 0.255).

## Discussion

### Seroprevalence of Hepatitis B Infection in HPUSM from 2014 to 2023

Hepatitis B is a significant global health burden, with Africa having the highest incidence of infection. The WHO Western Pacific Region and the WHO African Region account for approximately 97 million and 65 million chronically infected individuals, respectively (2). Understanding the local burden of hepatitis B infection is a critical step toward reaching the goal of hepatitis control and elimination by 2030, which aligns with WHO initiatives.

Malaysia has a medium seroprevalence of hepatitis B infection. Research conducted by the National Institutes of Health Malaysia has reported varying seroprevalence rates of hepatitis B across different populations in Malaysia, with a national prevalence range of 1.5% to 9.8% (5). In our study, we observed that the annual prevalence varied, ranging from 6.3% to 10.7%, with an overall seroprevalence of HBsAg of 9.1% (95% CI: 0.06, 0.12), with 28 of 308 patients testing positive. Our overall seroprevalence of 9.1% is consistent with the intermediate endemicity of hepatitis B infection in Malaysia (13).

Our study also showed an almost similar finding to a study conducted on the Negrito tribe, which included participants from five settlements in Kelantan and Perak; they found that the seroprevalence of HBsAg was 8.7% (14). However, the prevalence of HBsAg positivity in our study was higher than that in a previous study conducted among the Malaysian cohort (4%) and a recent extensive community campaign screening in Pahang (1.17%) (13, 15). The higher prevalence observed in our study could be attributed to several factors, with one of the main reasons being that our target patient population included individuals who were already receiving inpatient hospital care, including those with higher exposure risks, such as those with chronic health conditions or those who presented with hepatitis-related symptoms.

Additionally, geographical and socioeconomic status may also affect our study findings. HPUSM is in a region that may have a different epidemiological profile for HBV than other areas in Malaysia. Factors such as access to local healthcare, socioeconomic status and cultural practices can influence transmission dynamics.

### Sociodemographic Analysis of Patients Who Underwent HBsAg Screening at HPUSM

Our study provides insights into local sociodemographic factors, comorbidities, and health outcomes, as well as behavioural risks associated with the HBsAg positivity rate among patients screened at HPUSM between 2014 and 2023. Our study’s largest age group undergoing HBsAg screening was patients aged 45 years, representing 40.6% of the sample population. Interestingly, the highest HBsAg seropositivity rate was observed among patients in the 45 to 54 age group (14.3%), whereas patients over 64 had the lowest positivity rate (5.0%).

A similar trend was observed in a study from the Malaysia Cohort, which also reported a higher prevalence of HBsAg in their 45 to 54 age group, suggesting that this demographic is particularly vulnerable to HBV infection (13). Several factors, such as cumulative exposure to potential risk behaviours, historical vaccination practices, and socioeconomic factors, may contribute to the increased likelihood of chronic HBV infection in this age group. The introduction of the hepatitis B vaccination programme in Malaysia, which began in 1989, has led to significant progress with reductions in infection observed among younger populations, whereas older cohorts continued to exhibit higher seropositivity (16).

Our study observed that males had a significantly higher positivity rate (11.7%) compared with females (6.5%). This gender disparity in HBV infection is significant and consistent with other Malaysian studies. For instance, Lim et al. (15) found that males were more likely to test positive for HBsAg than females from community-based screening conducted from 2018 to 2019 in Pahang (15). Muhammad NA et al. (13) also reported that being male was significantly associated with a positive HBsAg test. These gender differences could be attributed to behavioural factors such as a higher rate of risky behaviours (e.g., intravenous drug use, multiple sexual partners) among men (13).

Most of the population screened in our study was Malay (92.2%), and the positivity rate among Malays was 9.2%. The positivity rate was 8.3% among non-Malays (7.8% of the study population). This disparity in ethnic distribution reflects the ethnic distribution of our study catchment area, which comprises more Malay individuals than the non-Malay population. However, our study differs from the results of a previous local study of the Malaysian cohort in which HBV infection was reported to be prevalent in certain ethnic groups, such as the Chinese, as well as the indigenous population of Sabah and Sarawak (13). A previous study proposed that specific ethnicities with their cultural practices and high-risk behaviours, such as unhygienic body tattooing, piercing, and sexual activities, may have contributed to the high prevalence of HBV in certain ethnicities.

Our analysis showed that only 6.8% of the population were working in the private sector, which was recorded as having the highest HBsAg seropositivity rate at 28.6%, followed by self-employed individuals at 11.5%. In Selangor, Rajamoorthy et al. also found a higher prevalence of HBsAg among private sector workers than among civil servants (17). The elevated positivity rate among private employees may be attributed to occupational exposure risks or other socioeconomic factors influencing susceptibility to hepatitis B infection, which can be further investigated in future studies.

Our data revealed that divorcees had the highest positive HBsAg test rate at 27.3%, despite comprising only 3.5% of the study population. This finding differs from those of other local studies, where most results recorded that the married group had a higher rate of HBsAg seropositivity (17, 18). The association between divorce and HBsAg positivity may be related to behavioural factors such as increased risk of exposure to infected partners. However, further research is needed to establish causal links and to understand the mechanism.

Our analysis showed that patients who had coinfection with hepatitis C virus (HCV) comprised 4.5% of the study population, with an HBsAg positivity rate of 21.4%. In contrast, the rate of coinfection with HIV was lower, at 2.6%, yet it exhibited a significantly higher HBsAg positivity rate of 62.5%. These findings highlight the critical relationship between coinfections and the prevalence of hepatitis B, suggesting that individuals with HIV are at greater risk for HBV infection than those with HCV. However, our study showed different statistics compared to a study by Akhtar et al. in a local tertiary hospital in Penang, where they found that the overall prevalence of hepatitis B positivity was much lower at a rate of 13% among their patients with HIV infection (18).

Our analysis also highlighted insights on chronic illness burden among patients with HBV infection that were postulated to facilitate progression into chronic liver disease. To date, there is no local study to compare the association of HBsAg positivity and comorbidities such as Type 2 diabetes mellitus (T2DM), hypertension, hyperlipidemia, and obesity that can further accelerate progression into a cirrhotic state. From our analysis, patients with T2DM constituted 32.5% of the study population and had an HBsAg positivity rate of 8.0%. Longstanding T2DM as a standalone disease already carries significant risk factors for advanced liver fibrosis (19). Apart from that, a cohort study from South Korea among healthy individuals found a significant association between hepatitis B infection and increased risk of diabetes incidents in the future (20). Our study also found that 42.2% of patients had hypertension, with an HBsAg positivity rate of 7.7%.

Meanwhile, 26.0% of our study populations had hyperlipidemia with an HBsAg positivity rate of 8.8%. Although obesity is a well-established risk factor for the development of chronic liver disease, mainly non-alcoholic fatty liver disease, our study found that patients with obesity, comprising 4.9% of the population, had the lowest HBsAg positivity rate among the examined comorbidities at 6.7%. A cross-sectional study in Southwest China showed that liver cirrhosis progression was more common among patients with hepatitis B infection with metabolic syndrome than without metabolic syndrome (21).

### Factors Associated with Hepatitis B Infection

This study assessed the factors associated with HBV infection among the study participants, highlighting specific risk factors and their impact on hepatitis B seroprevalence. The substantial association between needle sharing and hepatitis B infection was a key finding. In contrast, sex, ethnicity, family history of chronic hepatitis, blood transfusion history, multiple sexual partners, and body tattooing were not significant predictors of HBV infection.

Through simple logistic regression, our analysis clearly identified needle sharing as a significant risk factor for hepatitis B infection. The odds ratio (Adj. OR = 11.1; 95% CI: 2.12, 57.80; *P* = 0.004) underscores that individuals who engage in needle sharing have nearly nine times the odds of being seropositive for hepatitis B compared with those who do not share needles. This finding aligns with the existing literature, which consistently demonstrates that needle sharing is a high-risk behaviour for the transmission of bloodborne pathogens, such as hepatitis B, where the risk of transmission from a single hollow bore needle prick injury can be as high as 30% to 62% in HBeAg-positive individuals (22).

The significant association observed in this study emphasises the need for targeted harm reduction strategies and interventions to reduce needle sharing practices among at-risk populations. In contrast, Rajamoorthy et al. (23) conducted a local cross-sectional survey on hepatitis B-related risk behaviours among adults in Selangor and reported that only 1% of their study participants had a history of needle sharing. However, the study was limited by the fact that the survey was conducted among participants with unknown HBsAg status and did not directly confirm the presence of HBV infection among them (23).

In addition, our study explored the potential role of the association between sex and ethnicity in hepatitis B infection. Although females exhibited lower odds of seropositivity than males (Adj. OR = 0.62; 95% CI: 0.27, 1.42; *P* = 0.255), this difference was not statistically significant. Similarly, non-Malay patients had slightly higher odds of infection than Malays (OR = 1.11; 95% CI: 0.25, 4.98; *P* = 0.893), but this association also lacked statistical significance. These findings suggest that gender and ethnicity may not be independent strong predictors of HBV infection within our cohort. However, the non-significant findings do not entirely rule out potential differences in risk factors by gender or ethnicity, and further research with larger sample sizes might reveal more nuanced insights. Contrary to a local study involving more participants from the Malaysia Cohort, which found that HBsAg positivity was significantly associated with males and certain ethnic groups, such as the Chinese, as well as the indigenous populations of Sabah and Sarawak (13).

In addition, our study also examined the associations between hepatitis B infection and HIV infection and medical factors such as receiving blood transfusions or a family history of chronic hepatitis, as well as other high-risk behaviours such as multiple sexual partners and body tattooing. While blood transfusions and having multiple sexual partners increased the odds of infection, these associations were not statistically significant (OR = 1.86 for both factors). This could be attributed to the relatively small sample sizes of these subgroups or the low prevalence of these behaviours in our study population. Improvements in screening and safety measures for blood transfusions could have reduced the risk of HBV transmission through this route. Muhammad et al. (13) also reported that having a medical history of blood transfusion showed no significant association with HBsAg positivity.

There are a limited number of local studies to compare the significant associations between hepatitis B infection and family history of chronic hepatitis or exposure to high-risk behaviours such as multiple sexual partners and body tattooing. In support of our study, we found no significant association between the risk behaviour of having multiple sexual partners and hepatitis B infection. A cross-sectional study among pregnant women in Nigerian antenatal clinics also found no significant relationship between having multiple sexual partners and HBV infection transmission (24).

Our study showed a high HBsAg positivity rate of 100% among the small percentage (2.6%) of patients with a family history of chronic hepatitis. Logistic regression analysis revealed that having a family history of chronic hepatitis was associated with higher odds of having HBV infection. The substantial OR (22616647799.916) and wide CI indicated that the estimate was highly uncertain. Thus, having a family history of chronic hepatitis did not have a statistically significant association with hepatitis B infection (*P* = 0.999), as the logistic regression model could not reliably estimate the effect of this variable due to the small sample size and high uncertainty in the estimate. Our study differs from the findings of a study conducted among pregnant patients at public hospitals in Ethiopia, where a significant association was found between a family history of chronic hepatitis and hepatitis B infection positivity (*P* = 0.014) (25).

On another note, our analysis of the risk behaviour of body tattooing and hepatitis B infection showed no significant association, given that only one patient (0.3% of the study population) reported this exposure and tested positive for HBsAg, resulting in a 100% positivity rate among the group with a history of body tattooing. The standard error of this model was exceedingly large (S.E. 40,192.969), indicating extreme uncertainty in the estimate. This suggests that the model was not stable or reliable, likely because of the small sample size. In contrast to our study, Eke et al. reported a significant association between body tattooing (*P* = 0.001) and transmission of hepatitis B infection among antenatal Nigerian women (24).

Overall, this study’s results highlight that needle sharing has a significant impact on hepatitis B seroprevalence, and the need for targeted interventions to reduce this high-risk behaviour should be emphasised. Although family history or other risk factor behaviours, such as multiple sexual partners, history of blood transfusion, and body tattooing, were not statistically significant in our study, they are still relevant for understanding the broader risk landscape and may warrant attention in future research.

Several limitations were noted in this study. First, our study was based on a retrospective study design that relies on existing records that may contain missing, incomplete or inaccurate information. This can lead to biased or limited results because certain essential variables may not be recorded. Although probability sampling may reduce selection bias, a retrospective study is limited by historical or recorded data availability. Additionally, our study relied on reported data for some risk factors, which may lead to underreporting or inaccuracies. The small sample size for specific subgroups, such as those with a family history of chronic hepatitis or exposure to body tattooing, limits the reliability of the statistical analysis. Lastly, our study was conducted in a single institution, which may not represent the broader population, particularly given the low reported positivity of HBV infection in certain demographic groups.

Based on the findings of this study, several recommendations can be made to enhance the management and prevention of hepatitis B infection in the local region. First, there is a pressing need to increase awareness and education campaigns targeting high-risk populations, focusing on the dangers of needle sharing and the importance of safe injection handling. Additionally, to prevent new infections, local public health strategies should initiate and strengthen community-based vaccination programmes, especially among at-risk populations. Finally, further research with larger samples is warranted to explore the socioeconomic and cultural factors influencing HBV transmission in Malaysia, which could inform more tailored public health strategies.

## Conclusion

In conclusion, our study provides valuable insights into the epidemiology and burden of HBV infection among inpatients admitted to HPUSM from 2014 to 2023. The overall prevalence of HBsAg positivity was 9.1%, which is consistent with the intermediate endemicity of hepatitis B infection in Malaysia. Our study highlighted that the highest HBsAg positivity was observed in the 45- to 54-year age group, with a higher prevalence among males than females and a significant association between the risk behaviour of needle sharing and the transmission of hepatitis B infection in our local population.

## Figures and Tables

**Table 1:** Distribution of patients undergoing HBsAg testing at HPUSM over ten years, categorised by HBsAg status

Year of diagnosis	Total *n* (%)	Positive *n* (%)	Negative *n* (%)
2014	30 (9.7)	2 (6.7)	28 (93.3)
2015	31 (10.1)	3 (9.7)	28 (90.3)
2016	32 (10.4)	3 (9.4)	29 (90.6)
2017	32 (10.4)	3 (9.4)	29 (90.6)
2018	33 (10.7)	3 (9.1)	30 (90.9)
2019	30 (9.7)	3 (10.0)	27 (90.0)
2020	32 (10.4)	2 (6.3)	30 (93.8)
2021	28 (9.1)	3 (10.7)	25 (89.3)
2022	28 (9.1)	3 (10.7)	25 (89.3)
2023	32 (10.4)	3 (9.4)	29 (90.6)

**Table 2:** Distribution of HBsAg status across various demographic and medical history factors among patients in HPUSM from 2014 to 2023 (*N* = 308)

Variable	Total *n* (%)	Positive *n* (%)	Negative *n* (%)
**Age group (years old)**			

< 45	125 (40.6)	9 (7.2)	116 (92.8)
45 to 54	56 (18.2)	8 (14.3)	48 (85.7)
55 to 64	67 (21.8)	8 (11.9)	59 (88.1)
> 64	60 (19.4)	3 (5.0)	57 (95.0)

**Gender**			

Male	154 (50.0)	18 (11.7)	136 (88.3)
Female	154 (50.0)	10 (6.5)	144 (93.5)

**Ethnicity**			

Non-Malay	24 (7.8)	2 (8.3)	22 (91.7)
Malay	284 (92.2)	26 (9.2)	258 (90.8)

**Occupation**			

Civil servants	70 (22.7)	3 (4.3)	67 (95.7)
Private employee	21 (6.8)	6 (28.6)	15 (71.4)
Self-employed	87 (28.3)	10 (11.5)	77 (88.5)
Unemployed	119 (38.6)	8 (6.7)	111 (93.3)
Student	11 (3.6)	1 (9.1)	10 (90.9)

**Marital status**			

Single	47 (15.3)	5 (10.6)	42 (89.4)
Married	250 (81.2)	20 (8.0)	230 (92.0)
Divorcee	11 (3.5)	3 (27.3)	8 (72.7)

**Hepatitis C coinfection**			

No	294 (95.5)	25 (8.5)	269 (91.5)
Yes	14 (4.5)	3 (21.4)	11 (78.6)

**HIV coinfection**			

No	300 (97.4)	23 (7.7)	277 (92.3)
Yes	8 (2.6)	5 (62.5)	3 (37.5)

**Diabetes**			

No	208 (67.5)	20 (9.6)	188 (90.4)
Yes	100 (32.5)	8 (8.0)	92 (92.0)

**Hypertension**			

No	178 (57.8)	18 (10.1)	160 (89.9)
Yes	130 (42.2)	10 (7.7)	120 (92.3)

**Hyperlipidaemia**			

No	228 (74.0)	21 (9.2)	207 (90.8)
Yes	80 (26.0)	7 (8.8)	73 (91.2)

**Obesity**			

No	293 (95.1)	27 (9.2)	266 (90.8)
Yes	15 (4.9)	1 (6.7)	14 (93.3)

**Family history of chronic hepatitis**			

No	300 (97.4)	20 (6.7)	280 (93.3)
Yes	8 (2.6)	8 (100.0)	0 (0.0)

**History of receiving blood transfusion**			

No	281 (91.2)	24 (8.5)	257 (91.5)
Yes	27 (8.8)	4 (14.8)	23 (85.2)

**History of sharing a needle**			

No	302 (98.1)	25 (8.3)	277 (91.7)
Yes	6 (1.9)	3 (50.0)	3 (50.0)

**Multiple sexual partners**			

No	281 (91.2)	24 (8.5)	257 (91.5)
Yes	27 (8.8)	4 (14.8)	23 (85.2)

**History of having body tattoo**			

No	307 (99.7)	27 (8.8)	280 (91.2)
Yes	1 (0.3)	1 (100.0)	0 (0.0)

**Table 3:** Associated factor of hepatitis B infection seroprevalence among inpatients through simple logistic regression

Variables	OR (95% CI)	Wald statistic (*df*)	*P*-value
**Gender**			

Male	Ref.		
Female	0.53 (0.23, 1.18)	2.45 (1)	0.118

**Ethnicity**			

Malay	Ref.		
Non-Malay	1.11 (0.25, 4.98)	0.02 (1)	0.893

**History of blood transfusion**			

No	Ref.		
Yes	1.86 (0.60, 5.83)		

**Needle sharing**			

No	Ref.		
Yes	11.08 (2.12, 57.80)		

**Multiple sexual partners**			

No	Ref.		
Yes	1.86 (0.59, 5.81)		

**Table 4:** Associated factors of hepatitis B infection seroprevalence among inpatients through multiple logistic regression

Variable	Adj. OR (95% CI)	Wald statistic (*df*)	*P*-value
**Gender**			

Male	Ref.		
Female	0.62 (0.27, 1.42)	1.30 (1)	0.255

**Needle sharing**			

No	Ref.		
Yes	8.87 (1.64, 47.91)	6.43 (1)	0.011

Standard method was applied; No multicollinearity and no interaction; Classification table 90.9% Characteristics (ROC) curve was 60.1% correctlyclassified;
